# Case Report: Safety and efficacy of blinatumomab in combination with donor lymphocyte infusion for the prophylaxis of relapse following allogeneic hematopoietic stem cell transplantation in a pediatric patient with acute lymphoblastic leukemia

**DOI:** 10.3389/fimmu.2026.1825841

**Published:** 2026-06-17

**Authors:** Yi-Tong Li, Yu Li, Li-Na Wang, Li-Bin Huang

**Affiliations:** Department of Pediatrics, The First Affiliated Hospital, Sun Yat-sen University, Guangzhou, China

**Keywords:** allogeneic hematopoietic stem cell transplantation, blinatumomab, donor lymphocyte infusion, prophylaxis of relapse, refractory/relapsed acute lymphoblastic leukemia

## Abstract

**Background:**

Acute lymphoblastic leukemia (ALL) is the most common malignant hematological disease in children. Patients with high-risk ALL have a poor prognosis, and allogeneic hematopoietic stem cell transplantation (HSCT) is one of the important treatment modalities. However, post-transplant relapse of the primary disease remains one of the leading causes of treatment failure, seriously affecting the long-term survival of patients. Donor lymphocyte infusion (DLI) has been shown to have some efficacy in preventing relapse in B-cell ALL after HSCT, while the use of blinatumomab for preventing post-transplant relapse in ALL remains under investigation. Moreover, the safety and efficacy of the combination regimen for this indication still need to be verified in larger patient cohorts. We herein report a single case of a child with high-risk ALL who received blinatumomab combined with prophylactic DLI to prevent relapse after HSCT, along with a brief review of the relevant literature.

**Case presentation:**

A 5-year-old boy was diagnosed as ALL, early pro-B, KMT2A::USP2 fusion gene positive. After induction treatment with the SCCLG-ALL-2016 protocol, the minimal residual disease (MRD) by flow cytometry(FCM) and the quantitative PCR of KMT2A::USP2 fusion gene and NGS IGH remained continuous positive. The child received one cycle of blinatumomab, and the MRD by FCM and RQ-PCR turned negative after the blinatumomab regimen. Another two weeks of blinatumomab was given as bridge and then haploidentical HSCT was performed. Because his MRD by NGS IGH was 0.0311% before HSCT, the child received 3 cycles of blinatumomab and two prophylactic DLIs to prevent post-transplant relapse. During the treatment with blinatumomab and DLI, the child developed grade 1 cytokine release syndrome (CRS), without other severe adverse reactions such as neurotoxicity, hypoxemia, and hypotension.

**Conclusion:**

In this pediatric patient with high-risk KMT2A::USP2-positive ALL, sequential blinatumomab plus prophylactic DLI was well-tolerated and maintained sustained remission after HSCT. This case preliminary supports the combined regimen for post-transplant relapse prevention in high-risk patients. Given case report limitations, larger prospective trials are needed to verify its safety, efficacy and optimal dosing for widespread clinical use.

## Background

Acute lymphoblastic leukemia (ALL) is the most common malignancy in childhood. Significant progress has been made based on risk-stratified treatment strategies for pediatric ALL, with the long-term overall survival rate now exceeding 90%. However, approximately 20% of children still develop treatment resistance or experience relapse after achieving initial complete remission (1, 2).

Allogeneic hematopoietic stem cell transplantation (HSCT) serves as a pivotal therapeutic modality for refractory/relapsed ALL. Clinical studies have demonstrated that children with ALL undergoing HSCT after their first relapse achieve a long-term survival rate of approximately 50% (3). A large cohort study of 439 pediatric ALL revealed that the HSCT group had a significantly superior 5-year overall survival rate of 52.4% compared to the conventional chemotherapy group, with an event-free survival (EFS) rate of 51.7% (4).

Despite its established role as a cornerstone intervention for improving long-term outcomes in refractory/relapsed ALL, post-transplant relapse remains a formidable clinical challenge (5). The risk of relapse after HSCT is associated with MRD status before transplantation. The FORUM trial revealed that pediatric patients with ALL who had positive minimal residual disease (MRD≥10^-4^) before HSCT had a 3-year relapse rate of 33%, which was significantly higher than the 20% observed in MRD-negative patients (6). Currently, MRD assessment comprises multiparameter flow cytometry (mFCM), quantitative polymerase chain reaction (qPCR), and next-generation sequencing of immunoglobulin (NGS-Ig) gene rearrangements. Studies have indicated that positive NGS-Ig MRD status is the independent predictor of post-transplant relapse. Specifically, the 2-year relapse rate was 0% with an OS of 96% in the pre-transplant NGS-Ig MRD negative group, whereas the positive group had a markedly elevated relapse rate with an OS of only 77% (7).

Current strategies for preventing post-transplantation relapse in pediatric ALL encompass timely tapering or discontinuation of immunosuppressants, the use of targeted agents, prophylactic DLI, and early intervention guided by regular MRD monitoring. These approaches have evolved from reliance on conventional chemotherapy alone toward a multidimensional strategy integrating molecular monitoring, immunotherapy, targeted therapy, and cellular therapies (8–10). In recent years, immunotherapies and molecularly targeted treatments have achieved significant progress in managing refractory or relapsed ALL (10–12). Blinatumomab has demonstrated efficacy not only in efficiently eradicating MRD in cases with suboptimal responses to conventional chemotherapy or with relapsed/refractory disease, but also shows emerging potential as maintenance therapy following HSCT for high-risk ALL (13–16).A single-center phase II study (NCT02807883) reported that among 21 adult patients with high-risk B-ALL who received four cycles of blinatumomab maintenance post-HSCT, the 1-year OS rate was 85% (95% CI 61%-95%) and 1-year progression-free survival (PFS) rate was 71% (95% CI 47%-86%), with a favorable tolerability profile. With a median follow-up of 14.3 months, median OS and median PFS were not reached in this cohort; the cumulative incidence of relapse at 1 year was 29% (95% CI 11%-49%), and 1-year nonrelapse mortality (NRM) was 0% (17). However, the aforementioned evidence is primarily based on adult data, and published literature on the use of blinatumomab for post-transplant relapse prevention in the pediatric population is limited. Therefore, this article reports a case of a child with high-risk ALL who received blinatumomab combined with prophylactic DLI for relapse prophylaxis after HSCT, aiming to provide clinical insights and reference for hematologists and oncologists.

## Case presentation

A 5-year-old boy presented to our hospital’s Department of Oral and Maxillofacial Surgery on February 2, 2023, with left eye swelling and spontaneous unilateral knee ecchymosis. Tissue biopsy revealed B-lymphoblastic lymphoma/leukemia, leading to his referral to our department on February 16, 2023. Physical examination revealed mild edema of the left upper eyelid and left cheek. No enlarged superficial lymph nodes were palpable throughout the body. The liver was palpable 4 cm below the costal margin with a medium consistency, and the spleen was palpable 2 cm below the costal margin with a firm consistency. Initial complete blood count showed a white blood cell count (WBC) of 17.97×10^9^/L, neutrophils 2.70×10^9^/L, hemoglobin 99 g/L, and platelets 139×10^9^/L. Bone marrow smear demonstrated 63% blasts. Flow cytometry revealed 70.2% phenotypically abnormal immature B-lymphocytes, with an immunophenotype positive for HLA-DR, CD34, CD38, CD19, CD79a, CD56, IgVH, IgL, and TCRD. Chromosomal karyotype analysis showed 41∼46,XY, t(4;11) (q21;q23),der(16)t(1;16) (q21;q12),inc (4)/46,XY (16). Whole-exome sequencing and RNA sequencing (RNA-Seq) identified a KMT2A::USP2 fusion gene. Next-Generation Sequencing-Based Clonality Assessment of Ig Gene Rearrangements revealed significant clonality for IGH-SEQ1, IGH-SEQ2, IGK-SEQ3, and IGK-SEQ4, with clonal frequencies of 80%, 18.19%, 14.05%, and 6.63%, respectively. The patient had received no treatment prior to presentation at our department and had no relevant past medical history or family history of similar disorders. At initial diagnosis, the patient presented with an orbital mass. Sinus computed tomography (CT) revealed a lesion measuring 4.2×3.9×3.3 cm involving the left orbit. Histopathological biopsy confirmed B-lymphoblastic lymphoma/leukemia. Despite negative cerebrospinal fluid (CSF) cytology and flow cytometry, the patient was classified as central nervous system leukemia grade 3 (CNS3) due to the presence of impaired ocular mobility associated with orbital tumor infiltration.

**Figure 1:**
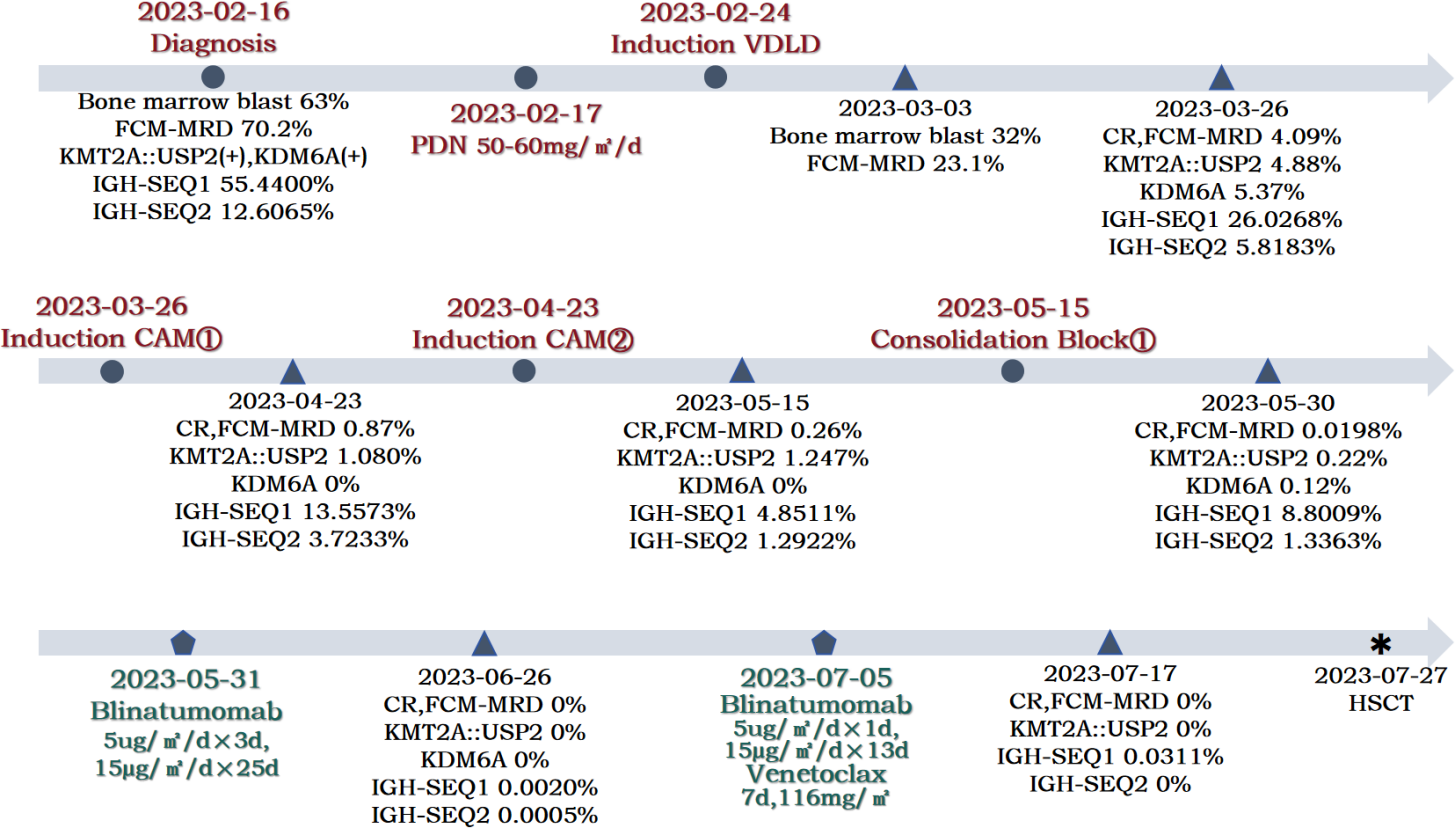
The schema of therapy in this patient.

**Figure 2:**
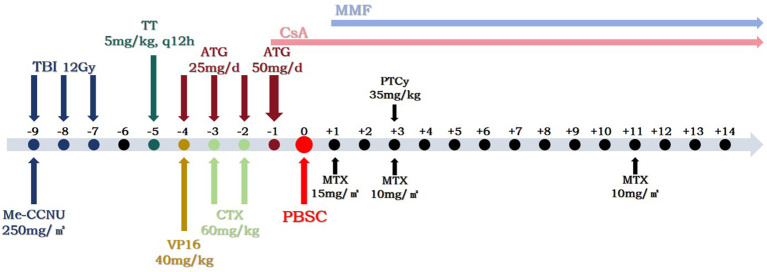
Process of pretreatment and transplantation.

Based on comprehensive medical history and ancillary investigations, the patient was diagnosed with early pro-B cell ALL, characterized by the presence of the KMT2A::USP2 fusion gene. Treatment was initiated using the SCCLG-ALL-2016 protocol, which is registered with the Chinese Clinical Trial Registry (ChiCTR; https://www.chictr.org.cn/; Registration Number: ChiCTR2000030357). After induction, MRD by FCM remained positive at 0.26%, RQ-PCR of KMT2A::USP2 was 1.247%, and NGS-Ig (IGH-SEQ1 4.8511%, IGH-SEQ2 1.2922%) were all continuously positive. (**Figure 1**). Given the high-risk cytogenetic feature of the KMT2A::USP2 fusion and persistent MRD positivity after induction-consolidation chemotherapy, HSCT was indicated to mitigate relapse risk and improve long-term survival. Informed consent was obtained from the patient and his family for the publication of this case report. After the first Block consolidation chemotherapy, A 28-day course of blinatumomab was administered from May 31 to June 28, 2023, with dose escalation from 5 μg/m²/d for the initial 3 days to 15 μg/m²/d for the subsequent 25 days, after which the MRD level decreased to less than 0.01%. Another two-week course of blinatumomab was administered from July 5 to July 19, 2023, as bridging therapy to further eliminate MRD prior to HSCT, with an initial dose of 5 μg/m²/d for 1 day followed by 15 μg/m²/d for the next 13 days, combined with 7-day venetoclax at a total daily dose of 116 mg/m². And then, a total body irradiation (TBI)-based conditioning regimen was administered from July 19 to July 21, 2023. The TBI was delivered via anterior-posterior (AP) and posterior-anterior (PA) fields at a prescribed dose of 12 Gy in 6 fractions (2 Gy twice daily), with shielding of the right eye lens, bilateral lungs, kidneys, bladder, and testes. The conditioning regimen further consisted of Me-CCNU (250 mg/m²), thiotepa (TT, 10 mg/kg), anti-thymocyte globulin (ATG, 7 mg/kg), etoposide (VP16, 40 mg/kg), and cyclophosphamide (CTX, 120 mg/kg/day). Before transplantation, the patient exhibited normal ocular mobility. Pre-transplant cranial PET-MRI revealed no soft-tissue mass or increased metabolic activity in the maxillary sinus, consistent with Deauville score 1. In addition, CSF cytology showed no leukemic blasts, and CSF flow cytometry was negative. On July 27, 2023, the patient underwent transplantation with peripheral blood stem cells from a related donor (father, haploidentical, blood group O to O). The graft contained 8.3×10^8^/kg mononuclear cells, with an infused CD34^+^ cell dose of 14.3×10^6^/kg. Both neutrophil and platelet engraftment were achieved by day +13 post-transplantation (**Figure 2**). During the HSCT course, complications included engraftment syndrome, grade III oral mucositis, infectious fever, upper respiratory tract infection, and multiple electrolyte imbalances, all of which were ultimately resolved with appropriate management. Graft-versus-host disease (GVHD) prophylaxis included PTCy, short-course methotrexate (MTX), cyclosporine A (CsA), and mycophenolate mofetil (MMF). PTCy was given as a single dose of 35 mg/kg on day +3 after HSCT. MMF was discontinued on day +31 post-transplant, and CsA was tapered and stopped on day +89 post-transplant.

In this case, the pediatric patient had persistently positive NGS-Ig prior to transplantation, placing him in a high-risk group for post-transplant relapse; thus, active measures should be taken for relapse prophylaxis. Therefore, immunosuppressive agents including cyclosporine were gradually tapered and discontinued around month +3. The first DLI, with a CD3+ cell dose of 8.9×10^5^/kg, was administered on September 25, 2023(HSCT d+60). The first post-transplant cycle of blinatumomab was initiated on October 25, 2023 (HSCT d.

**Figure 3:**
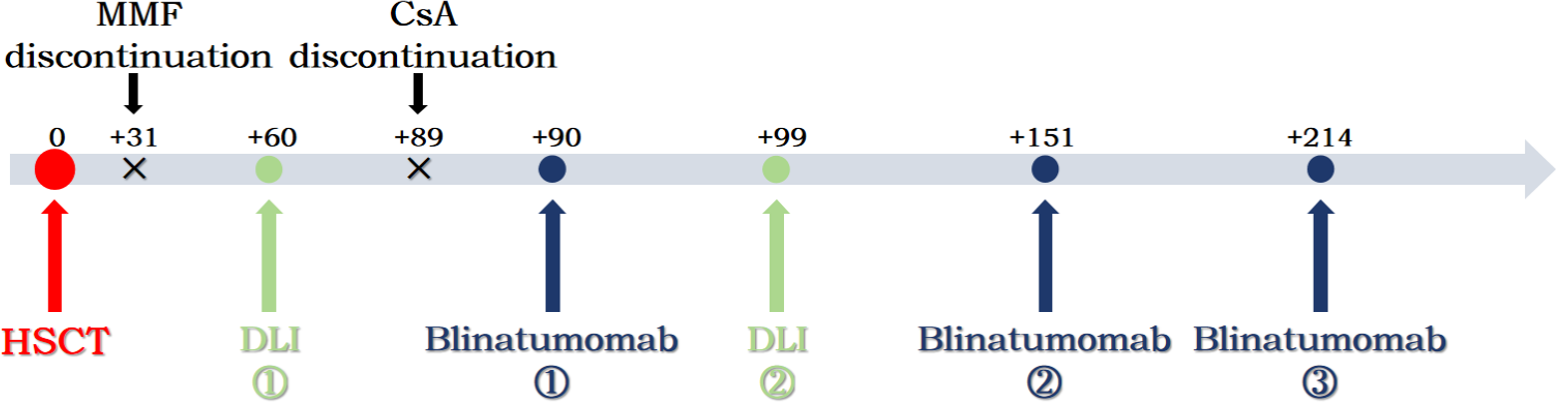
The post-transplant interventions.

**Figure 4:**
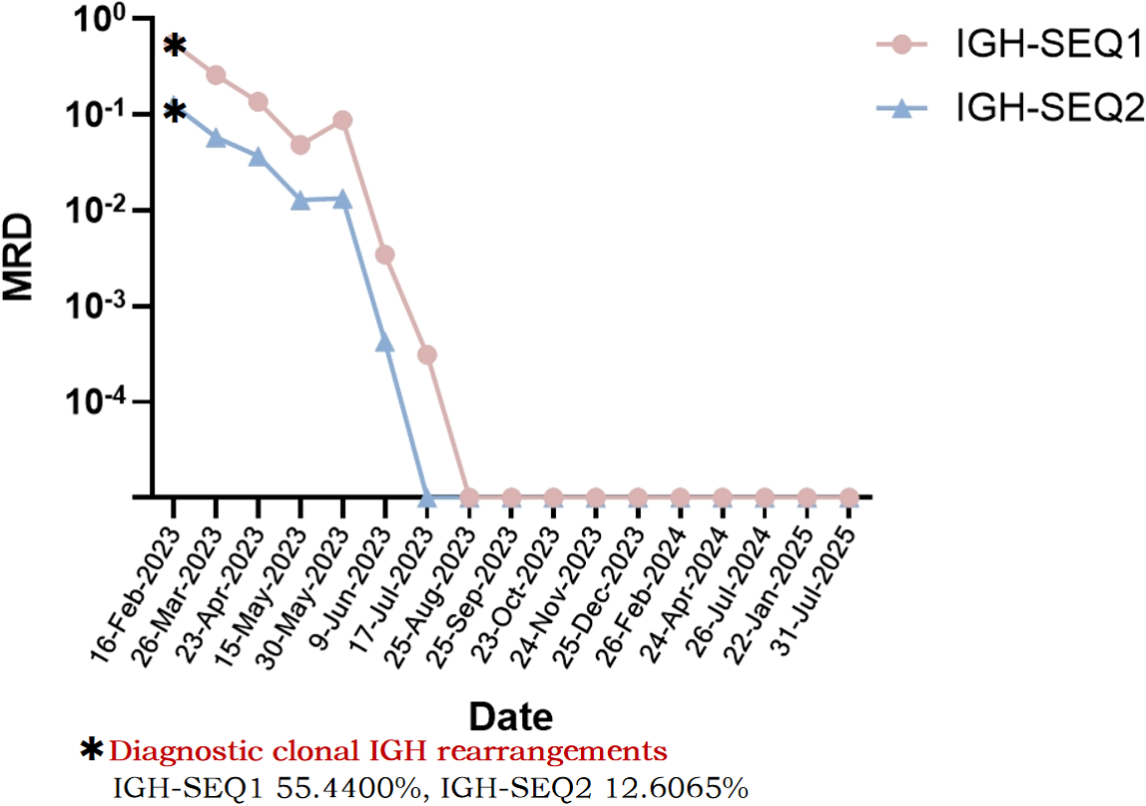
The trend of NGS IGH rearrangement.

+90; 5 µg/m²/day for 3 days, then 10 µg/m²/day for 3 days, followed by 15 µg/m²/day for 10 days). During this cycle, a second DLI (CD3^+^ cell dose approximately 5×10^6^/kg) was given on November 3, 2023 (HSCT d+99; day 8 of blinatumomab). On the day following the second DLI, the patient developed a pruritic rash on the back, which resolved with antihistamine treatment. No other signs of graft-versus-host disease were observed, and the rash was considered likely related to the DLI. Subsequent maintenance cycles of blinatumomab were administered on December 25, 2023 (HSCT d+151; 5 µg/m²/day for 2 days, then 10 µg/m²/day for 1 day, followed by 15 µg/m²/day for 12 days) and February 26, 2024 (HSCT d+214; 5 µg/m²/day for 3 days, then 15 µg/m²/day for 11 days). Post-transplant interventions are shown in **Figure 3**. During blinatumomab treatment, the patient experienced grade 1 CRS, which was managed supportively and resolved. As shown in **Figure 4**, bone marrow MRD remained negative at all time-points when DLI or blinatumomab was administered.

Dynamic changes of immune cell subsets and serum IgG levels after transplantation are summarized in **Table 1**. A decline in IgG levels was observed following blinatumomab administration. The proportion of Treg cells increased during each cycle of blinatumomab treatment and decreased after drug discontinuation. The variation trend of the CD4^+^/CD8^+^ T cell ratio was unpredictable in this case.

**Table 1:** Immune reconstitution indicators after HSCT.

Date	CD4^+^T cell (%)	CD8^+^T cell (%)	Treg (%)	IgG (g/L)
2023-07-27 HSCT				
2023/8/28	19.2	70.2	-	8.13
2023-09-25 DLI (1)				
2023/9/25 pre-DLI	9.3	81	-	7.15
2023/10/23	10.4	71.9	-	5.86
2023-10-25 Blinatumomab (1)				
2023/10/30	15.66	74.85	3.5	–
2023-11-03 DLI (2)				
2023/11/6	17.7	68.6	7.78	4.33
2023/11/10	19.1	56.6	9.04	-
2023-12-25 Blinatumomab (2)				
2023/12/25 pre-Blinatumomab	17.97	61.91	4.26	3.05
2024/1/10	27.86	57.4	6.4	2.65
2024-02-26 Blinatumomab (3)				
2024/2/26 pre-Blinatumomab	25.86	47.75	5.34	2.22
2024/3/13	28.56	58.04	7.12	-
2024/4/25	35	43.4	-	2.59
2024/7/29	30.5	42.5	-	5.53
2025/1/23	35.9	37.4	-	5.8
2025/7/31	36.4	33	-	8.79

To date, post-transplant monitoring shows sustained complete remission: bone marrow morphology is consistent with complete remission, MRD by FCM was negative, the quantitative levels of KMT2A::USP2 fusion gene and KDM6A mutation were 0%, and Ig-NGS MRD was below the detection limit. (<10^-6^, **Figure 4**). Serial post-transplant bone marrow chimerism monitoring demonstrated sustained full donor chimerism, with donor chimerism levels fluctuating between 99.44% and 99.82%. Regarding central nervous system leukemia, serial cranial MRI after transplantation showed no evidence of disease progression, and no deterioration of ocular function was observed.

**Figure 5:**
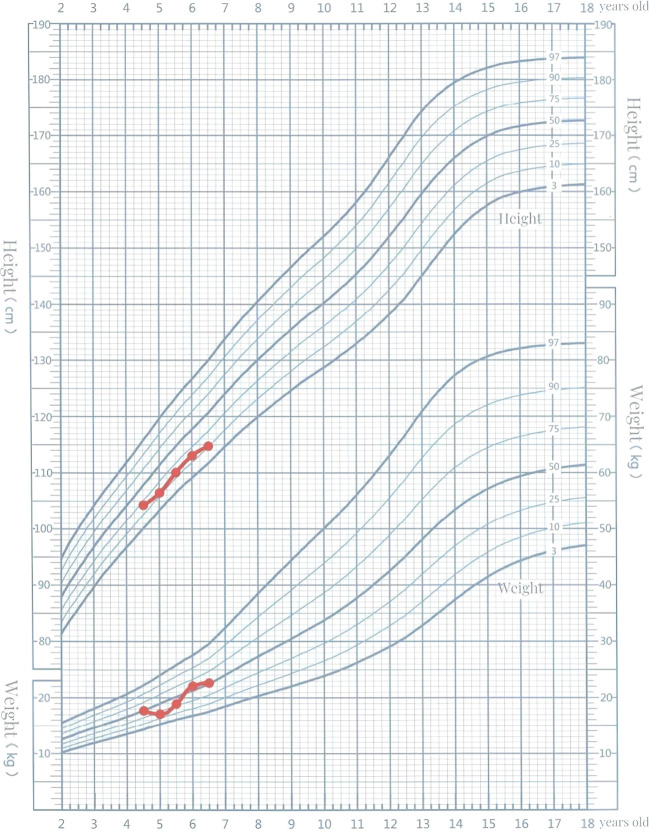
The growth and development status after HSCT.

Monitoring of organ function in the child is detailed in **Table 2**. With organ shielding during TBI (as applied in the conditioning regimen), gonadal reserve was relatively preserved; renal and thyroid function remained within normal limits. Serial orbital MRI and pulmonary function tests showed no abnormalities, with no evidence of TBI-related organ damage. Tracking of growth and development is detailed in **Figure 5**. After the child underwent HSCT, the body weight was at the 50th percentile of the growth curve for children of the same age, and the height was approximately at the 25th percentile of the growth curve for children of the same age, with a stable growth trajectory. These findings suggest that physical development after transplantation maintained a normal growth rate, without obvious growth retardation or nutritional imbalance.

**Table 2:** Organ function indicators after HSCT.

Date	InhibinB (pg/ml)	AMH (ng/ml)	FSH (IU/L)	LH (IU/L)	T (ng/ml)	TSH (uIU/mL)	T3 (pmol/L)	T4 (pmol/L)	CREA (umol/L)	eGFR (ml/min.1.73 m^2^)
2023/12/26	35	31.36	1.19	0.05	<0.13	2.539	4.74	10.06	23	166.63
2024/7/26	11	63.98	1.45	0.07	<0.13	6.23	7.61	10.04	21	190.84
2025/1/23	89	77.48	0.95	0.04	<0.13	2.857	8.46	11.77	22	187.15
2025/7/30	50	92.03	1.14	0.05	<0.13	3.354	6.93	12.03	22	189.97

## Discussion

DLI is a critical intervention for managing post-HSCT relapse or refractory hematological malignancies. Its underlying mechanism involves harnessing the immune reactivity of donor-derived T cells to eradicate residual leukemic cells. Studies indicate that prophylactic or preemptive DLI following allogeneic HSCT for pediatric hematologic malignancies is associated with favorable 5-year event-free survival rates. However, the risk of GVHD correlates with the infused cell dose; patients receiving dose-escalation regimens show a trend toward reduced incidence of moderate-to-severe chronic GVHD (borderline significance, p=0.051) (18). The efficacy of DLI is influenced by multiple factors, including tumor burden, timing of relapse, donor T-cell predominance, and cell dose. DLI demonstrates more pronounced effects in settings of low tumor burden and early-stage relapse (19). In the present case, DLI was administered prophylactically while the patient was in sustained bone marrow remission. This approach aimed to leverage the significant anti-leukemic activity of DLI during the early post-transplant phase when tumor burden is minimal.

Blinatumomab, a bispecific T-cell engager antibody, exerts its therapeutic effect by simultaneously binding to the CD19 antigen on B-ALL cells and the CD3 antigen on T cells. This dual binding effectively recruits and activates cytotoxic T lymphocytes, triggering a targeted immune response that leads to the elimination of leukemic cells. In the treatment of pediatric refractory or relapsed (R/R) ALL, its clinical utility is well-established and varies according to the phase of therapy in which it is applied. Prior to HSCT, blinatumomab serves as an effective bridging or consolidative therapy, creating a window for successful transplantation. In a phase I/II study (NCT01471782), blinatumomab administered at 15 µg/m²/day demonstrated a favorable tolerability profile in 49 children with R/R B-ALL. Among them, 39% achieved complete remission (CR) with single-agent therapy (20). The subsequent RIALTO expanded access study (NCT02187354) further confirmed its efficacy, reporting that 63% of pediatric patients achieved CR within the first two treatment cycles, with 83% of responders attaining MRD negativity (14, 21). Furthermore, two randomized trials (NCT02393859 and NCT02101853) indicated that blinatumomab, when used as consolidation therapy prior to allo-HSCT, significantly improved EFS in children with high-risk first-relapse B-ALL compared to conventional chemotherapy. It also improved RFS and OS in standard-risk patients (22, 23). In the present case, the patient exhibited persistently positive MRD by flow cytometry following high-risk induction, VDLD-CAM, and consolidation with BLOCK(1) chemotherapy. After receiving one cycle of blinatumomab, the patient achieved MRD negativity in the bone marrow, which successfully enabled bridging to HSCT.

As a salvage therapy for post-transplant relapse, blinatumomab has demonstrated promising efficacy. A study involving 38 pediatric patients with post-HSCT relapse reported an overall response rate of 34% following blinatumomab treatment, with 24% achieving sustained molecular remission, thereby creating an opportunity for a second transplantation (24). Furthermore, preliminary data support its use for relapse prevention after transplantation. A single-center phase II study evaluating blinatumomab maintenance in post-transplant B-ALL patients (median age 30 years) showed a 1-year OS rate of 85% and a 1-year PFS rate of 71%, with a favorable tolerability profile (17). Similarly, a study in a pediatric post-transplant setting observed a relapse rate of 20% in patients receiving blinatumomab maintenance, which was significantly lower than the 31% rate in the control group without maintenance therapy (25). These findings suggest that post-transplant administration of blinatumomab can effectively control residual disease and reduce the risk of relapse.

Both DLI and blinatumomab can be utilized for relapse prevention following transplantation in ALL. This raises the question of whether combining these two modalities could yield superior outcomes. In recent years, both blinatumomab and DLI have demonstrated potential as maintenance therapies to prevent relapse after HSCT. They are thought to improve long-term survival and quality of life by enhancing the patient’s immune response and reducing tumor burden (13, 17, 26). Blinatumomab functions by forming a lytic synapse between T cells and tumor cells, leading to tumor cell lysis and apoptosis. Based on this mechanism, we postulated that administering DLI on day +8 of a blinatumomab cycle might lead to a synergistic antitumor effect (27, 28). The infused donor lymphocytes from DLI can augment the host immune system. These cells may synergize with the blinatumomab-activated T cells, potentially enabling more effective recognition and clearance of residual leukemic cells. Concurrently, blinatumomab reduces tumor burden through direct T cell-mediated cytotoxicity, which may create a more favorable microenvironment for the efficacy of DLI.

The effects of maintenance treatment with blinatumomab combined with DLI on post-transplant immune reconstitution remain poorly elucidated. Studies have demonstrated that immune reconstitution occurs gradually following HSCT. In the early post-transplant period, the proportion of CD4^+^ T cells remains low, while CD8^+^ T cells undergo dominant expansion, resulting in an inverted CD4/CD8 ratio (29). Based on the sequential immunotherapeutic strategy adopted in this case, DLI exogenously supplies mature functional T cells. On one hand, it facilitates the early expansion of donor-derived CD8^+^ cytotoxic T cells and lays the foundation for graft-versus-leukemia (GVL) effects. On the other hand, it optimizes the immune microenvironment and promotes CD4^+^ T cell reconstitution after transplantation. Meanwhile, blinatumomab sustains the cytotoxic activity of CD8^+^ T cells and reduces MRD, further constructing a favorable immune microenvironment to potentiate the GVL effect mediated by DLI. After two cycles of DLI treatment, the proportion of CD8^+^ T cells gradually declines alongside the progressive recovery of CD4^+^ T cells and eventually reaches a steady state, indicating satisfactory cellular immune reconstitution. In addition, the patient achieved sustained disease remission with low tumor burden after HSCT. No obvious proliferation of CD4^+^and CD8^+^ T cells was observed during blinatumomab administration, which is consistent with previous literature findings (30).

Serum IgG levels decreased during combined treatment with DLI and blinatumomab. This phenomenon is attributed to targeted depletion of B cells and incomplete recovery of helper function of CD4^+^ T cells. Subsequent recovery of cellular immunity contributes to the gradual elevation of IgG levels, suggesting progressive restoration of humoral immune function.

The proportion of regulatory T cells (Treg) is relatively low in the early post-transplant phase, which is consistent with the inherent delayed reconstitution characteristics of Treg cells (31). Two rounds of DLI markedly elevate Treg proportions, which is jointly induced by infused donor lymphocytes and endogenous compensatory proliferation. Elevated Treg cells can effectively suppress excessive activation of alloreactive T cells. The dynamic alteration pattern of Treg cells under blinatumomab treatment has not been fully clarified. In this case, Treg proportions increased during three courses of blinatumomab therapy and declined after drug withdrawal. The upregulation of Treg cells may restrain excessive inflammatory responses and protect hematopoietic and immune reconstitution. Notably, the absence of a remarkable reduction in Treg levels does not indicate DLI treatment failure. The gradual recovery of CD4^+^ T cells, restored CD4/CD8 ratio and late elevation of IgG collectively confirm favorable immune reconstitution. Further investigations with expanded case cohorts are required to explore the comprehensive impacts of combined blinatumomab and DLI therapy on immune reconstitution.

Regarding safety, blinatumomab is associated with a relatively low incidence of adverse events and offers a more favorable safety profile compared to conventional chemotherapy (24). The standard treatment protocol recommends continuous infusion over four weeks per cycle. However, in our center, a modified administration strategy was employed when used post-HSCT. Following an initial slow dose escalation starting at 5 µg/m², a target dose of 15 µg/m² was administered via continuous infusion, with treatment cycles averaging 14 days.

In this case, the patient developed grade 1 CRS manifesting as low-grade fever and localized rash during blinatumomab treatment, which resolved after symptomatic management with antipyretics and intensified local skin care. No blinatumomab-related neurotoxicity was observed; thus, prophylactic levetiracetam was not administered. Notably, no other severe adverse events including central nervous system abnormalities, hypoxemia or hypotension occurred, and such favorable tolerability may be attributed to the adopted dose-escalation strategy.

Furthermore, the rash that developed during the second DLI in this case necessitated differentiation between an infusion-related reaction and DLI-induced GVHD. Consequently, DLI therapy requires careful balancing of the GVL effect against the risk of GVHD, with dose and schedule adjustments guided by individual treatment response. While immunosuppressants are routinely administered post-transplantation to prevent GVHD, they concurrently suppress donor T-cell activity, thereby potentially diminishing the GVL effect of DLI. Therefore, in this patient, immunosuppressive agents were gradually tapered and discontinued around month +3 post-transplantation. Additionally, DLI was omitted during the third post-transplant cycle of blinatumomab due to prior mild GVHD. Throughout the follow-up period, no clinical manifestations of GVHD were observed.

The combined approach adopted in this case offers a feasible strategy for relapse prevention following transplantation. Future investigations into blinatumomab plus prophylactic DLI therapy should focus on elucidating the optimal sequence and timing of the combination, as well as its interaction with immunosuppressive agents. These aspects warrant validation in larger cohorts and more in-depth mechanistic exploration.

## Conclusions

The combination of blinatumomab and prophylactic DLI appears to be a safe and potentially effective strategy for preventing relapse in acute lymphoblastic leukemia following allogeneic hematopoietic stem cell transplantation, representing a potential prophylactic option in the post-transplant setting. To date, the pediatric patient in this case has achieved sustained complete remission, resulting in improved survival outcomes.

## Data Availability

The datasets presented in this study can be found in online repositories. The names of the repository/repositories and accession number(s) can be found in the article/supplementary material.

## References

[1] Pehlivan KC Duncan BB Lee DW . CAR-T cell therapy for acute lymphoblastic leukemia: transforming the treatment of relapsed and refractory disease. Curr Hematol Malig Rep. (2018) 13:396–406. doi: 10.1007/s11899-018-0470-x 30120708

[2] Lv M Liu Y Liu W Xing Y Zhang S . Immunotherapy for pediatric acute lymphoblastic leukemia: recent advances and future perspectives. Front Immunol. (2022) 13:921894:. doi: 10.3389/fimmu.2022.921894 35769486 PMC9234114

[3] Hunger SP Raetz EA . How I treat relapsed acute lymphoblastic leukemia in the pediatric population. Blood. (2020) 136:1803–12. doi: 10.1182/blood.2019004043 32589723

[4] Morales D Loth G Colturato VAR Tavares RB Lermontov S Rodrigues AM . Outcomes of hematopoietic stem cell transplantation in pediatric acute lymphoblastic leukemia: a multicenter Brazilian cohort study. Bone Marrow Transplant. (2025) 60:1344–50. doi: 10.1038/s41409-025-02676-1 40707781

[5] Oskarsson T Söderhäll S Arvidson J Forestier E Montgomery S Bottai M . Relapsed childhood acute lymphoblastic leukemia in the Nordic countries: prognostic factors, treatment and outcome. Haematologica. (2016) 101:68–76. doi: 10.3324/haematol.2015.131680 26494838 PMC4697893

[6] Balduzzi A Glogova E Peters C Sedlacek P Dalle JH Locatelli F . Impact of minimal residual disease on the outcome of hematopoietic stem cell transplantation for childhood acute lymphoblastic leukemia within the FORUM trial. Haematologica. (2026) 111:122–34. doi: 10.3324/haematol.2025.287456 40820816 PMC12775767

[7] Pulsipher MA Carlson C Langholz B Wall DA Schultz KR Bunin N . IgH-V(D)J NGS-MRD measurement pre- and early post-allotransplant defines very low- and very high-risk ALL patients. Blood. (2015) 125:3501–8. doi: 10.1182/blood-2014-12-615757 25862561 PMC4447864

[8] Talleur AC Naik S Gottschalk S . Preventing relapse after CD19 CAR T-cell therapy for pediatric ALL: the role of transplant and enhanced CAR T cells. Hematol Am Soc Hematol Educ Program. (2023) 2023:91–6. doi: 10.1182/hematology.2023000424 38066941 PMC10727085

[9] Fang P Gao Y Xin H Liu L Liu Y Xu Y . Efficacy of WT1 gene-guided pre-emptive therapy for prevention of relapse in acute myeloid leukemia after transplantation and its optimal intervention threshold. Zhong Nan Da Xue Xue Bao Yi Xue Ban. (2024) 49:1120–9. doi: 10.11817/j.issn.1672-7347.2024.240351 PMC11495973 39788500

[10] Li X Zhang J Liu F Liu T Zhang R Chen Y . Olverembatinib treatment in pediatric patients with relapsed Philadelphia-chromosome-positive acute lymphoblastic leukemia. Clin Lymphoma Myeloma Leuk. (2023) 23:660–6. doi: 10.1016/j.clml.2023.04.012 37301632

[11] Hall AG Rau RE . Blinatumomab use in pediatric B-ALL: where are we now? Blood Adv. (2025) 9:3946–54. doi: 10.1182/bloodadvances.2024014043 40489801 PMC12337177

[12] Pan J Tang K Luo Y Seery S Tan Y Deng B . Sequential CD19 and CD22 chimeric antigen receptor T-cell therapy for childhood refractory or relapsed B-cell acute lymphocytic leukaemia: a single-arm, phase 2 study. Lancet Oncol. (2023) 24:1229–41. doi: 10.1016/s1470-2045(23)00436-9 37863088

[13] Huang J Shi B Yu S Xue M Wang L Jiang J . Efficacy of blinatumomab as maintenance therapy for B-lineage acute lymphoblastic leukemia/lymphoma following allogeneic hematopoietic cell transplantation. Blood Cancer J. (2024) 14:109. doi: 10.1038/s41408-024-01092-w 38977689 PMC11231304

[14] Rau RE Gupta S Kairalla JA Rabin KR Angiolillo A Wang C . Blinatumomab added to chemotherapy improves disease-free survival in newly diagnosed NCI standard risk pediatric B-acute lymphoblastic leukemia: results from the randomized Children's Oncology Group Study AALL1731. Blood. (2024) 144:1. doi: 10.1182/blood-2024-207450 38963672

[15] Qi P Zhang Y Wu Y Yu J Fan J Lin W . Efficacy and safety of adding blinatumomab to first-line treatment for Chinese children with B-cell acute lymphoblastic leukemia. Blood. (2024) 144:312. doi: 10.1182/blood-2024-199937

[16] Ji J Liu Z Chen X Kuang P Dong T Zhang H . Mini-dosed blinatumomab maintenance therapy following allogeneic hematopoietic stem cell transplantation in acute B-cell lymphoblastic leukemia. Blood. (2024) 144:1049. doi: 10.1182/blood-2024-210268

[17] Gaballa MR Banerjee P Milton DR Jiang X Ganesh C Khazal S . Blinatumomab maintenance after allogeneic hematopoietic cell transplantation for B-lineage acute lymphoblastic leukemia. Blood. (2022) 139:1908–19. doi: 10.1182/blood.2021013290 34914826 PMC8952188

[18] Hou MH Lee CY Ho CY Yu TY Hung GY Huang FL . Donor lymphocyte infusion for prophylaxis and treatment of relapse in pediatric hematologic Malignancies after allogeneic hematopoietic stem cell transplant. J Chin Med Assoc. (2023) 86:991–1000. doi: 10.1097/jcma.0000000000000992 37697465 PMC12718955

[19] Toprak SK . Donor lymphocyte infusion in myeloid disorders. Transfus Apher Sci. (2018) 57:178–86. doi: 10.1016/j.transci.2018.04.018 29754984

[20] von Stackelberg A Locatelli F Zugmaier G Handgretinger R Trippett TM Rizzari C . Phase I/Phase II study of blinatumomab in pediatric patients with relapsed/refractory acute lymphoblastic leukemia. J Clin Oncol. (2016) 34:4381–9. doi: 10.1200/jco.2016.67.3301 27998223

[21] Locatelli F Zugmaier G Mergen N Bader P Jeha S Schlegel PG . Blinatumomab in pediatric relapsed/refractory B-cell acute lymphoblastic leukemia: RIALTO expanded access study final analysis. Blood Adv. (2022) 6:1004–14. doi: 10.1182/bloodadvances.2021005579 34979020 PMC8945309

[22] Locatelli F Zugmaier G Rizzari C Morris JD Gruhn B Klingebiel T . Effect of blinatumomab vs chemotherapy on event-free survival among children with high-risk first-relapse B-cell acute lymphoblastic leukemia: a randomized clinical trial. Jama. (2021) 325:843–54. doi: 10.1001/jama.2021.0987 33651091 PMC7926287

[23] Hogan LE Brown PA Ji L Xu X Devidas M Bhatla T . Children's Oncology Group AALL1331: phase III trial of blinatumomab in children, adolescents, and young adults with low-risk B-cell ALL in first relapse. J Clin Oncol. (2023) 41:4118–29. doi: 10.1200/jco.22.02200 37257143 PMC10852366

[24] Queudeville M Schlegel P Heinz AT Lenz T Döring M Holzer U . Blinatumomab in pediatric patients with relapsed/refractory B-cell precursor acute lymphoblastic leukemia. Eur J Haematol. (2021) 106:473–83. doi: 10.1111/ejh.13569 33320384

[25] Breviglieri CNM Gouveia RV Ginani VC Santos CN de Oliveira MRS Batalha ABW . Post-stem cell transplant maintenance for pediatric acute leukemias: insights from a Brazilian institution with a Latin American perspective. Front Oncol. (2025) 15:1540158:. doi: 10.3389/fonc.2025.1540158 40110204 PMC11919910

[26] Abematsu T Nishikawa T Kasabata H Nakagawa S Okamoto Y . Blinatumomab maintenance therapy following bone marrow transplantation for early relapsed pediatric B-cell precursor acute lymphoblastic leukemia and analysis of lymphocyte subset changes. Cureus. (2024) 16:e62263. doi: 10.7759/cureus.62263 39006644 PMC11245324

[27] Yang L Lai X Yang T Lu Y Liu L Shi J . Prophylactic versus preemptive modified donor lymphocyte infusion for high-risk acute leukemia after allogeneic hematopoietic stem cell transplantation: a multicenter retrospective study. Bone Marrow Transplant. (2024) 59:85–92. doi: 10.1038/s41409-023-02137-7 37907756

[28] Ueda M de Lima M Caimi P Tomlinson B Little J Creger R . Concurrent blinatumomab and donor lymphocyte infusions for treatment of relapsed pre-B-cell ALL after allogeneic hematopoietic cell transplant. Bone Marrow Transplant. (2016) 51:1253–5. doi: 10.1038/bmt.2016.104 27088374

[29] Hammoudi T Nucera S Troullioud Lucas AG Ansari M Balduzzi A Bertaina A . Harmonized immune recovery monitoring after HCT: evidence and practical guidance from the Westhafen Intercontinental Group. Blood Adv. (2025) 9:6141–57. doi: 10.1182/bloodadvances.2025016915 40902074 PMC12719163

[30] Ocadlikova D Lussana F Fracchiolla N Bonifacio M Santoro L Delia M . Blinatumomab differentially modulates peripheral blood and bone marrow immune cell repertoire: a Campus ALL study. Br J Haematol. (2023) 203:637–50. doi: 10.1111/bjh.19104 37700538

[31] Blazar BR MacDonald KPA Hill GR . Immune regulatory cell infusion for graft-versus-host disease prevention and therapy. Blood. (2018) 131:2651–60. doi: 10.1182/blood-2017-11-785865 29728401 PMC6032895

